# Longitudinal association of homocysteine with depressive and anxiety symptoms among urban adults: healthy aging in neighborhoods of diversity across the life span study

**DOI:** 10.1038/s41398-024-03111-7

**Published:** 2024-10-19

**Authors:** Michael F. Georgescu, May A. Beydoun, Christian A. Maino Vieytes, Marie T. Fanelli-Kuczmarski, Jason Ashe, Hind A. Beydoun, Sharmin Hossain, Nicole Noren Hooten, Michele K. Evans, Alan B. Zonderman

**Affiliations:** 1 Laboratory of Epidemiology and Population Sciences, National Institute on Aging Intramural Research Program, Baltimore, MD 21224 USA; 2 VA National Center on Homelessness Among Veterans, U.S. Department of Veterans Affairs, Washington, DC 20420 USA; 3 Department of Management, Policy, and Community Health, School of Public Health, University of Texas Health Science Center at Houston, Houston, TX 77030 USA; 4 Department of Human Services, State of Maryland, Baltimore, MD 21202 USA

**Keywords:** Human behaviour, Predictive markers, Physiology

## Abstract

Longitudinal associations of homocysteine (HCY) with depressive symptoms scores among urban adults remain under-studied, especially across sex, race and levels of anxiety. We examined longitudinal associations of homocysteine (HCY) with depressive symptoms scores among urban adults, before and after stratifying by sex, race and anxiety level, using data from 1460 Healthy Aging in Neighborhoods of Diversity across the Lifespan Study (HANDLS) participants aged 30–64 y at v_1_ (2004–2009), followed across 3 visits up to 2017. In addition to LnHcy_v1_, we used group-based trajectory models predicting z-transformed likelihood of greater LnHcy with age (Hcy_traj_). Total and domain-specific depression symptoms were scored using Center for Epidemiologic Studies Depression (CES-D) scale. Mixed-effects linear regression models and Cox proportional hazards models were utilized. A positive association was found between baseline LnHcy_v1_ and CES-D total scores in reduced socio-demographic- adjusted Model 1 (β (standard error [SE]) = + 2.337 (0.902), *P* = 0.010), a relationship slightly attenuated in fully adjusted Model 2 (Model 1 adjusting for lifestyle and health factors) with a β (SE) = + 1.825 (0.883), *P* = 0.039. Individuals with lower anxiety levels experienced faster CES-D domain 2 score annualized increase over time (interpersonal problems) with higher LnHcy_v1_ (*β* (SE) = 0.041 (0.018), *P* = 0.024). Hcy_traj_ was linked to incident elevated depressive symptoms (CES-D total score ≥16) overall (fully adjusted model: HR = 1.09, 95% CI: 1.03–1.14, *P* = 0.001), particularly among women and those living in poverty. Baseline and “high trajectory” of LnHcy were positively associated with depressive symptoms and elevated depressive symptom incidence, in a sex-, race-, poverty status- and anxiety-level specific manner.

## Introduction

According to the World Health Organization and United Nations Development Program projections, the number of those over 60 would increase to 2.1 billion by 2050, from 1.4 billion in 2030 [1, 2]. Declines in social, cognitive, and physical function associated with aging has been related to worse quality of life [3]. An earlier study discovered a reciprocal association between quality of life in older age and mental health outcomes, such as anxiety and depression [4]. Depression, while being underdiagnosed frequently, is a major contributor to the worldwide sickness burden and has been linked to higher rates of morbidity and mortaltiy in the elderly [2, 5]. Hu et al.‘s latest 48-study meta-analysis estimated that 28.4% of older adults had depression, with a 95% confidence interval (CI) ranging from 24.8% to 32.0% [1]. Personal characteristics associated with geriatric depression include female sex, aging, being single or divorced, having less education, being unemployed, low income, no health insurance, smoking, having grown up through traumatic experiences, low self-esteem, social deprivation, being alone or lonely, bereavement, chronic conditions, cognitive impairment, poor health, and history of depression [2].

Studies suggest that stressful life experiences and long-term chronic stress may be linked to geriatric depression [6–8]. Physical symptoms are more common in late life onset depression than in early onset depression, and vascular disease may contribute to, exacerbate, or prolong depressive symptoms in old age [5]. A meta-analysis of 61 prospective cohort studies found that the presence of late-life depression may raise the risk of cardiovascular disease-related death by >30% [5]. Investigating connection between stressors and symptoms of depression can help identify strategies for averting late-life depression and its detrimental impact on health. Homocysteine (HCY), which plays a crucial role in the biochemical balance within the central nervous system, shows strong associations with cardiovascular (i.e., stroke and coronary artery disease), neurological (i.e., dementia), and psychiatric disease (depression and anxiety) when elevated [9–22]. A meta-analysis of 46-studies revealed that elevated HCY is associated with depression [23]. Several studies identified positive relationships between elevated HCY levels and anxiety disorders, including PTSD [13, 17, 18, 24]. However, despite such evidence, findings concerning HCY’s associations with depressive symptoms, in the presence or absence of anxiety disorders remains minimal.

In this study, urban middle-aged adults participating in the Health Aging in Neighborhoods of Diversity across the Life Span (HANDLS) investigation had their long-term associations between HCY and depression examined. We hypothesized that, differently depending on race and sex, higher HCY would be associated with higher symptoms of anxiety and depression. We also investigated whether anxiety levels affected the relationship over time between HCY and depressive symptoms. Both anxiety and depressive symptoms were compared to the long-term levels and change in HCY with age as a secondary analysis.

## Methods

### Database

Initiated in 2004 by the National Institute on Aging (NIA) Intramural Research Program (IRP), the HANDLS study is a prospective cohort study conducted to address research topics linked to health disparities in age-associated disorders. Using an area probability sampling strategy, middle-aged African American and White individuals of both sexes (baseline age: 30-64 years) were chosen from thirteen Baltimore city neighborhoods with widely varying household incomes in order to create the sample of urban adults for the HANDLS study [25]. Additionally, to increase participation rates and retention among non-traditional research participants, the HANDLS project makes use of mobile medical research vehicles (MRVs) and innovative research methods. The HANDLS project was authorized by the National Institutes of Health’s Institutional Review Board, and participants gave written informed consent [25].

Between 2004 and 2009, baseline (Visit 1) data on HANDLS participants were gathered in two stages: (i) an in-home interview including health status, health care utilization, psychosocial factors, diet, neighborhood characteristics, and demographics comprised the first phase of the study and (ii) the subsequent stage encompassed the following aspects: medical history, physical examination, dietary recall, cognitive assessment, psychophysiological evaluations (e.g., heart rate variability, arterial thickness, carotid ultrasonography, muscle strength, bone density assessments), and laboratory assessments (e.g., blood chemistries, hematology, biomarkers of oxidative stress, biomaterials for genetic studies) all carried out in MRVs. Following the initial visit, HANDLS participants were contacted every five years for Visits 2 and 3, which were conducted between 2009 and 2013 and 2013 and 2017, respectively. HANDLS data documentation are available at https://handls.nih.gov/06Coll-w00dataDocR.cgi. While some HANDLS study visits involved distinct forms of evaluations, several assessments were repeated throughout time. This analysis harnessed data from Visits 1, 2, and 3, including data on blood HCY concentrations and depressive symptom scores.

### Measures

#### Homocysteine (HCY)

Plasma HCY was measured by the Alinity i analyzer Aeon Technologies, LLC (Frostburg, MD). First, the serum quality was measured by hemolysis, lipemia, and icteria detection. All serum samples passed the quality check threshold determined by Aeon Technologies. HCY was quantified using the Alinity i Homocysteine assay on the Alinity i analyzer [26]. This assay is a one-step immunoassay that uses chemiluminescent microparticle immunoassay (CMIA) technology. The analytical measuring range (AMR) of the Alinity i Homocysteine assay is 1.00 to 50.00 mmol/L (0.14 to 6.76 mg/mL). Samples were run in 12 batches and a serum sample (Cat # 200-0162; Stem Cell Technologies) was run in each batch as a control. The homocysteine values for this serum control ranged from 4.55 to 6.18, with an inter-assay coefficient of variation of 8.38%.

Using a STATA plugin (*traj* and *trajplot*) modified from a well-established SAS approach [27, 28], group-based trajectory modeling (GBTM) was carried out for Log_e_ transformed HCY, or LnHcy measured at visits 1, 2, and 3. This allowed for the identification of adult groups with comparable developmental trajectories in terms of level and trends throughout time. This group-based method uses maximum likelihood and a multinomial modeling strategy to estimate model parameters. The quasi-Newton method was selected as the likelihood optimization technique. The *traj* command uses maximum likelihood estimation to preserve data and mitigate biases from listwise elimination, assuming missingness at random.

Group-based trajectories with age were displayed with 95% confidence intervals (CI) for each group trajectory. We defined a censored normal distribution for LnHcy, with intercept (0), linear (1), or quadratic (2) orders for each group trajectory, as appropriate. The most parsimonious model with best the fit was determined using the BIC. If linearity was established using the *trajplot*, up to three groups were taken into consideration, and the linear model was selected. Group membership probability was the main metric of interest obtained from the GBTM. If there was an identified trajectory group with less than 10% prevalence in the three-group model, then two groups were selected. Given prior evidence, the trajectory group with elevated and/or increasing HCY over time was selected as the main exposure of interest [9–22], and the exposure was operationalized using group membership probability.

#### Depressive symptoms

Using self-reported information from the Center for Epidemiological Studies Depression (CES-D) questionnaire, a depressed symptoms score was computed [29]. Several samples of older adults have demonstrated the adequate psychometric properties of the CES-D questionnaire (e.g. [30]). With 20 items and item scores ranging from “0” to “3,” the CES-D total score range anywhere from “0” and “60.” The frequency and intensity of depressed symptomatology during the previous week were the main aspects of the CES-D questions. Participants in the HANDLS study were asked to select whether they experienced an item most or all of the time (score = 3), seldom or a substantial portion of the time (score = 2), rarely or never (score = 0), or some or a little of the time (score = 1). Some questions required reverse coding. Additionally, we looked at domain-specific CES-D scores, such as those for (1) depressive affect (such as feeling depressed); (2) interpersonal problems (such as experiencing social anxiety); (3) somatic complaints (such as difficulty sleeping or eating); and (4) positive affect (such as thinking positively) [29]. By summing the scores for depressed symptoms for each item under each domain, we were able to determine the raw CES-D sub-scores for each domain. Information about the items used to calculate each domain-specific CES-D sub-score is described elsewhere [29]. A binary version of CES-D total score was also computed using a cutoff of 16, commonly used in other studies [31], for a sensitivity analysis on elevated depressive symptoms (EDS) incidence.

#### Anxiety symptoms

Self-report information from the Psychiatric Diagnostic Screening Questionnaire Generalized Anxiety Disorder subscale (PDSQga) was used to compute an anxiety symptom score. There were strong psychometric properties ascribed to the PDSQga among adults [32, 33]. There are a total of 10 items with a scoring of “No”, “Yes”, “Don’t Know”, “Not Applicable”. A total PDSQga score ≥ 6 indicated substantial anxiety symptoms. Information about the items used to calculate anxiety symptoms is described in Supplementary Method 1.

For a secondary analysis whereby anxiety symptoms were the outcome of interest, GBTM was used to obtain two groups for anxiety scores between visits 1 and 3 (High vs. Low), using a zero-inflated Poisson (zip) distribution. This binary trajectory outcome was then converted into a Log_e_(odds of “High” anxiety) and used as main continuous outcome of interest in a series of multiple linear regression models [27, 28]. Anxiety disorder was also elicited at all 3 visits. This variable was used for validation only by cross-tabulating it with the main anxiety disorder variable (above vs. below median) and examining mean Log_e_(odds of “High” anxiety) across baseline anxiety disorder.

##### Covariates

The 2010 Healthy Eating Index [HEI-2010] and health (body mass index [BMI; weight/height2 in kg.m^−2^, continuous]) characteristics were among factors taken into account when examining the hypothesized relationships between HCY and depressive symptoms. Other key confounders included demographics (sex (male, female), age (years), race (White, African American), poverty status (<125% federal poverty line, ≥125% federal poverty line), education (less than high school, high school, more than high school), lifestyle (current cigarette smoking [Yes, No]) and use of drugs (Yes, No [using any of marijuana, opiates, and cocaine]). The age at baseline (Visit 1) was examined as a continuous variable, and the follow-up time durations were computed using the age at Visits 2 and 3. The Department of Health and Human Services’ poverty standards were used to operationalize the state of poverty based on household income and total household size [34]. The HEI-2010 [34] based its overall diet quality recommendations on food and macronutrient standards for the American population from the Dietary Guidelines for Americans. We also described the relationship between HCY and several downstream health-related factors including self-rated health and comorbidities without including those into the main models given their potential mediating effect between HCY and depressive symptoms. Comorbidities were characterized as follows: self-reported history of any of several cardiovascular diseases (no, yes), including atrial fibrillation, angina, coronary artery disease, congestive heart failure, and myocardial infarction; diabetes (non-diabetic, pre-diabetic, diabetic); dyslipidemia ([or statin use] no, yes); and hypertension (no, yes). Three categories for self-rated health were established: very good/excellent, good, and poor/average.

### Effect modifiers

The main effect modifiers in this study were sex (male vs. female) and race (African American vs. White). In addition, baseline anxiety symptoms (above vs. below median of total score) was also considered as an important effect modifier in the association between HCY and depressive symptoms over time.

### Statistical methods

Stata version 18 (StataCorp, College Station, TX) was used to conduct descriptive, bivariate, and multivariable analyses. While counts and percentages were employed to describe categorical data, measures of central tendency (mean, median) and dispersion (standard deviation, standard error, interquartile range) were used to characterize continuous variables. Testing for multicollinearity among the variables included in mixed-effects models using correlation matrices was one of the model-building procedures. The *mixed* command in Stata removes missing data from analysis by removing observations with missing outcomes, but can cause data loss and biases if not random. Nevertheless, the model includes observations with at least one outcome available, assuming missingness at random. Given that each covariate had less than 5% missing data on average, we ensured sample sizes were constant between different adjusted models by performing multiple imputations (5 imputations, 10 iterations) using the chained equations methodology in order to reduce missing data caused by the addition of covariates into different models. Only the potentially confounding covariates were imputed, while outcome and exposures were not. Stata commands to this end included *mi impute*, *mi passive* and *mi estimate* among others. During this estimation method, all covariates were entered simultaneously, and continuous covariates were centered on their means, just like in earlier research [35, 36]. First, using the largest sample after excluding HANDLS subjects with missing CES-D data, baseline sociodemographic, lifestyle, and health characteristics, CES-D test scores (at baseline and change over time), as well as LnHcy_v1_ and z-transformed probability of higher LnHcy with age, were described before and after stratifying according to Hcy_v1_ tertiles. Differences across tertiles were tested using a series of bivariate linear regression and multinomial logistic regression models to compare means and odds of belonging to specific categories across these tertiles. Specifically, the uppermost two tertiles of LnHcy_v1_ were compared to the lowest tertile. Second, different sets of covariates were accounted for when building a series of mixed-effects linear regression models for baseline HCY as a predictor of CES-D test scores (at baseline and change over time) and z-transformed probability of higher HCY trajectory as a predictor of CES-D test scores (at baseline and change over time), (Supplementary method 2). Time spent on study, measured in years, between visits 1 and 3 was the time variable employed. Age, sex, race, poverty status, inverse mills ratio (IMR), time spent on studies, and its interaction with variables including LnHcy_v1_ and Hcy_traj_ alternative exposures were all taken into account while adjusting *Model 1*. Model 2 included time on study and its interaction with LnHcy_v1_ or Hcy_traj_ and other covariates such as age, sex, race, poverty status, education, literacy, smoking, drug use, and the 2010-HEI, BMI, and IMR. For Models 1 and 2, the interaction effects of LnHcy_v1_ or Hcy_traj_ with sex and race were assessed as a sensitivity analysis. Moreover, stratified analyses were carried out independently for White and African American HANDLS participants, males, and women, as was done in previous studies examining blood biomarkers in relation to depressive symptoms [31, 37–39]. Therefore, we applied Models 1-2 to two exposures (LnHcy_v1_ and Hcy_traj_), five CES-D test scores (one total score and four domain-specific scores), two stratifying variables (sex, race), and up to two repeats (impact on baseline CES-D test scores and effect on change in CES-D test scores). Using a two-stage Heckman selection technique, we corrected for sample selectivity resulting from missing data in all models [26]. We estimated those mixed-effects linear regression models adjusted for the IMR in addition to the aforementioned covariates after using a probit regression model to predict an indicator of selection with sex, age at Visit 1, race, and poverty status. This model produced an IMR, or a function of the probability of being selected given these characteristics [40]. Predictive margins from the main mixed-effects linear regression models (Model 1, CES-D total score) were obtained and plotted across time for illustration purposes.

As a secondary outcome, the global anxiety score was treated similarly, but with a maximum of two visits (visits 1 and 3). In contrast to the CES-D score outcome, Log_e_ odds(“high anxiety trajectory”) was the main outcome of interest which was entered into a linear regression model. Prior to multiple testing correction, the type I error rate for the main and interaction effects was predetermined to be 0.05 and 0.10, respectively [41]. We used the familywise Bonferroni correction approach [42] to adjust for outcome multiplicity (i.e., five CES-D test scores and one global anxiety test score), especially for Model 1. Following that, Model 2 was regarded as a sensitivity model that incorporated variables that could be mediating or confounding. In line with earlier research [43], we therefore changed the significance thresholds for the main effects to *p* < 0.010 (0.05/5) and the two-way interaction terms to 0.10/5 = 0.020.

Four sensitivity analyses were also performed. First, Ln(odds of elevated anxiety) was the outcome in a linear regression model with primary predictor being baseline Log_e_(Hcy) [LnHcy]. A reduced and a fully adjusted model was conducted. Second, mixed-effects linear regression models were carried out whereby the main outcomes were CES-D total and domain-specific scores and the predictor was annualized change in LnHcy between visits 1 and 2. The models were incrementally adjusted as in earlier models. Moreover, a third sensitivity analysis was conducted whereby a mixed-effects linear regression model with time-dependent LnHcy as the outcome was conducted in the overall sample examining its association with various exposures, including baseline CES-D total score, ordinal anxiety score, probability of higher anxiety score and anxiety disorder at baseline. Finally, Cox proportional hazards models were conducted on the time-dependent binary version of the CES-D total score with a cutoff of 16. Time on study was used as the time variable. Analyses were also conducted on multiple imputed data. Specifically, the fully adjusted models were presented, examining associations of baseline and trajectory exposures of Hcy with incidence of EDS, after adjustment for the same vector of covariates included in the mixed-effects linear regression models. Stratification by sex, race, poverty status and anxiety score level was carried out, while also testing for interaction between each exposure and effect modifier separately in the fully adjusted models. The full Stata script will be made available on github at: https://github.com/baydounm/HANDLS_Hcy_Depression_Anxiety.

## Results

**Fig. 1:**
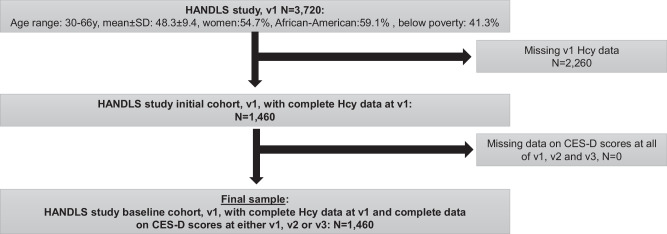
Study Flowchart – HANDLS (2004-2017). Notes: CES-D Center for Epidemiological Studies Depression, HANDLS healthy aging in neighborhoods of diversity across the lifespan study.

**Fig. 2:**
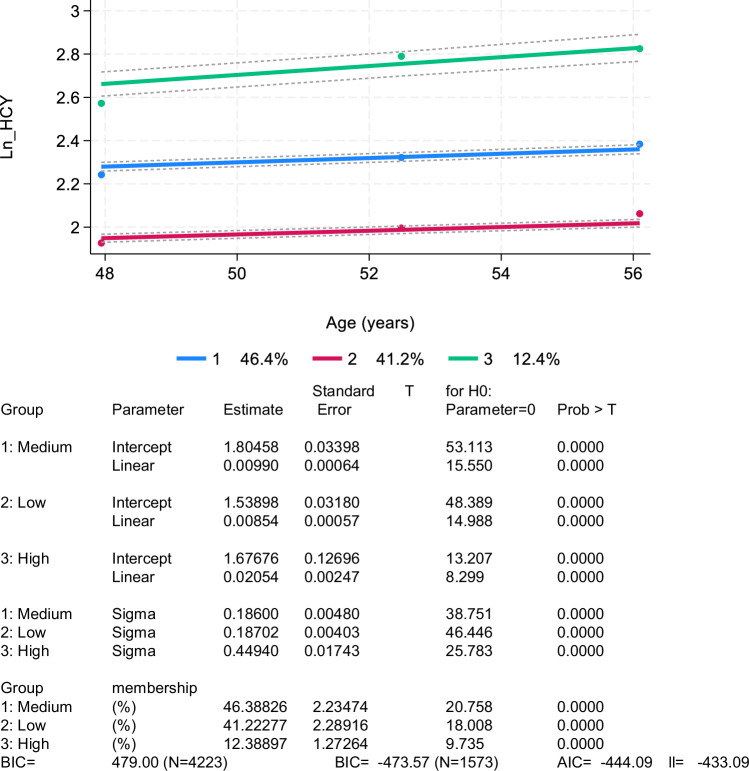
Group-based trajectories for homocysteine – HANDLS (2004-2017). Notes: HANDLS healthy aging in neighborhoods of diversity across the lifespan study; A = Table display of intercept and linear terms for the three trajectories in homocysteine identified using group-based trajectories; B = A graphical display of the three groups identified using group-based trajectory modeling is shown, whereby Ln_Hcy represents homocysteine total score (Log_e_ transformed) and Age (years) represents the time variable.

**Fig. 3:**
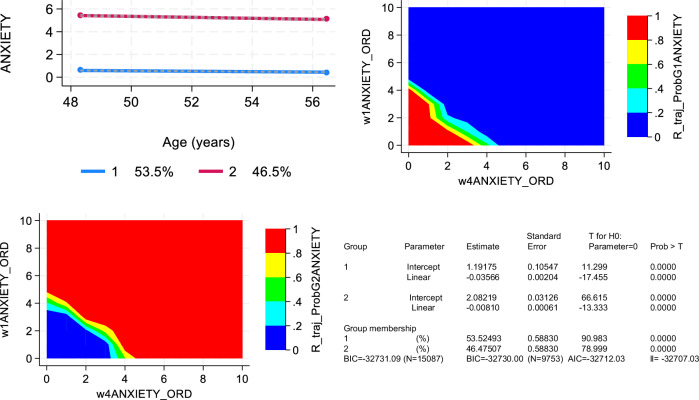
Group-based trajectories for anxiety score – HANDLS (2004–2017). Notes: HANDLS healthy aging in neighborhoods of diversity across the lifespan study; A = Table display of intercept and linear terms for the three trajectories in anxiety score, ordered (ANXIETY) identified using group-based trajectories; B = A graphical display of the two groups identified using group-based trajectory modeling is shown, whereby ANXIETY is the ordered anxiety score (range:0–10) and Age (years) represents the time variable.

As shown in Fig. 1, of 3,720 participants recruited initially in the HANDLS study, 1,460 participants had complete data on Hcy at visit 1, all of whom had complete data on CES-D scores at any of visits 1, 2 or 3, which made the final analytic sample for the main outcome of depressive symptoms over time. Based on Fig. 2, three separate groups were identified using group-based trajectory modeling for LnHcy levels, indicating a positive linear trend with age and an initial level that can be labelled as “Low”, “Medium” and “High”. The probability of belonging to the “High” group was on average 0.12. Importantly, 1 SD in Hcyt_raj_ in the selected sample was equivalent to a 0.28 increased probability of membership in the “High” group (data shown on https://github.com/baydounm/HANDLS_Hcy_Depression_Anxiety). Figure 3 shows the findings for the anxiety score using GBTM, which yielded two groups, namely “High” (46.5%) vs. “Low” (53.5%).

Table 1 describes socio-demographic, lifestyle and health characteristics at baseline as well as HCY and CES-D total scores (at baseline and change over time), overall, and across tertiles of baseline HCY. The means ( ± standard error [19]) for baseline LnHcy and CES-D total scores were estimated at 2.15 ( ± 0.01) and 14.01 ( ± 0.29), respectively. Similarly, the means ( ± SEM) for probability of high LnHcy and between-visit change in CES-D total score (annualized empirical bayes estimator) were estimated at 0.12 ± 0.01 and −0.12 ± 0.00, respectively. Means and proportions of several characteristics differed across baseline HCY tertiles. Mean age increased across the tertiles of Hcy as did the prevalence proportions of hypertension, diabetes, and elevated cholesterol. In contrast, we observed lower HEI-2010 scores, proportions of female subjects, and proportions of cigarette smokers across higher tertiles of HCY. Non-linear J-shaped associations between Hcy tertiles and CES-D total and sub-domain scores were detected. Similarly, ordered baseline anxiety score exhibited a non-linear inverted U-shaped association with HCY tertiles and an inverse relationship with the Log-odds of “high anxiety” probability.

**Table 1:** Summary statistics for socio-demographic, lifestyle and health characteristics at baseline (visit 1: 2004–2009); homocysteine, depressive symptoms and anxiety scores (at baseline and change over time), overall, and according to tertiles of baseline homocysteine (*N* = 1460): HANDLS 2004–2017.

	% or Mean ± SEM	Hcy at visit 1 tertiles		
	*N* = 1460	*n* = 485	*n* = 489	*n* = 486
		1st	2nd	3rd
**Homocysteine:**				
Hcy at visit 1	9.18 ± 0.14	6.29 ± 0.04	8.42 ± 0.03***	12.82 ± 0.35***
LnHcy at visit 1	2.15 ± 0.01	1.83 ± 0.01	2.13 ± 0.00***	2.49 ± 0.01***
Hcy_traj_: probability of “High” LnHcy	0.12 ± 0.01	0.02 ± 0.00	0.05 ± 0.01*	0.29 ± 0.02***
Hcy_change_: annualized change between visits 1 and 2	0.25 ± 0.04	0.30 ± 0.02	0.21 ± 0.03	0.25 ± 0.13
**Socio-demographic:**				
Sex, %:				
Male	42.4	24.1	43.0***	60.5***
Female	58.0	76.0	57.5	40.0
Age (years):				
Continuous	47.92 ± 0.24	45.73 ± 0.42	48.30 ± 0.42***	49.73 ± 0.39***
Race, %:				
White	43.2	44.3	44.0	42.0
African American	57.0	56.0	56.4	58.4
Poverty status, %:				
< 125% federal poverty line	37.0	37.3	36.4	37.0
≥ 125% federal poverty line	63.1	63.0	64.0	63.0
Education:				
Less than high school	6.2	6.1	6.0	7.0
High school	57.0	56.0	56.0	59.0
More than high school	37.2	38.3	38.5	35.0
**Lifestyle:**				
Cigarette smoking:				
Yes	43.5	40.0	44.0	20.1
No	57.0	60.2	56.5	80.0
Drug use:				
Yes	18.2	15.1	19.5	47.2
No	82.0	85.0	81.0	53.0
HEI-2010 score:	43.11 ± 0.33	44.34 ± 0.63	43.34 ± 0.59	41.66 ± 0.50**
HEALTH:				
Body mass index (kg/m^2^):	29.89 ± 0.19	30.08 ± 0.34	29.99 ± 0.34	29.61 ± 0.33
Self-rated health:				
Poor/Average	21.0	19.2	19.0	25.0
Good	39.1	39.2	39.5	39.0
Very good/Excellent	40.0	42.0	42.0	36.3
Hypertension:				
Yes	40.1	33.0	42.0**	46.0*
No	60.0	67.2	58.3	54.0
Diabetes:				
None	68.1	72.2	69.0	63.0
Pre-diabetes	18.0	15.0	18.1	21.0**
Diabetes	14.0	13.0	13.2	16.0
High Cholesterol:				
Yes	24.0	21.0	23.2	27.3
No	76.2	79.1	77.0	73.0
Cardiovascular disease:				
Yes	14.5	16.0	14.0	14.2
No	86.0	84.4	86.4	86.0
**Depressive symptoms:** ^**a**^				
Visit 1				
CES-D total score (*n* = 1445)	14.01 ± 0.29	14.34 ± 0.51	13.20 ± 0.49	14.50 ± 0.53*
CES-D domain 1 score (*n* = 1449)	4.34 ± 0.13	4.51 ± 0.22	4.03 ± 0.21	4.49 ± 0.23*
CES-D domain 2 score (*n* = 1449)	0.91 ± 0.03	0.95 ± 0.06	0.88 ± 0.06	0.89 ± 0.06*
CES-D domain 3 score (*n* = 1449)	6.42 ± 0.12	6.56 ± 0.20	6.09 ± 0.20	6.61 ± 0.21*
CES-D domain 4 score (*n* = 1449)	9.66 ± 0.07	9.69 ± 0.12	9.80 ± 0.12	9.50 ± 0.12*
CES-D total score ≥ 16, %	36.6	36.9	35.3	37.7
Visit 1 to Visit 3				
**Anxiety:**				
Anxiety total score, ordered (*n* = 1181)	2.78 ± 0.09	2.78 ± 0.15	2.94 ± 0.17*	2.62 ± 0.16*
*Z*-scored probability “High anxiety” trajectory (*n* = 1383)	−0.05 ± 0.03	−0.03 ± 0.05	−0.02 ± 0.05	−0.11 ± 0.05
“High anxiety” Trajectory probability (*n* = 1383)	0.44 ± 0.01	0.45 ± 0.02	0.46 ± 0.02	0.41 ± 0.02
Log-odds of “High anxiety” trajectory (*n* = 1224)	−1.10 ± 0.21	−0.62 ± 0.36	−0.98 ± 0.38	−1.73 ± 0.37*
Anxiety total score, below and above median (*n* = 1181), %				
Below median	52.0	49.0	51.0	55.4
Above median	48.3	51.3	49.2	45.0
Anxiety Disorder (*n* = 1346), %				
Yes	11.0	11.5	12.0	9.3
No	89.2	89.0	88.4	91.0
**Depressive symptoms annualized rate of change, visits 1 through 3:** ^**a**^				
CES-D total, empirical bayes estimator	−0.12 ± 0.00	−0.12 ± 0.01	−0.11 ± 0.00	−0.13 ± 0.01*
CES-D domain 1, empirical bayes estimator	−0.07 ± 0.00	−0.07 ± 0.00	−0.07 ± 0.00	−0.07 ± 0.00
CES-D domain 2, empirical bayes estimator	−0.01 ± 0.00	−0.01 ± 0.00	−0.01 ± 0.00	−0.01 ± 0.00
CES-D domain 3, empirical bayes estimator	−0.08 ± 0.00	−0.08 ± 0.00	−0.07 ± 0.00	−0.08 ± 0.00
CES-D domain 4, empirical bayes estimator	−0.05 ± 0.00	−0.05 ± 0.00	−0.06 ± 0.00*	−0.04 ± 0.00

Statistically significant association: *p < 0.05, **p < 0.01, ***p < 0.001, using *t*-tests from bivariate linear and multinomial logit regression models with Hcy tertile as the only predictor, comparing the two upper tertiles of Hcyv1 with lowest Hcyv1 tertile.

*CES-D* Center for Epidemiological Studies Depression, *Hcy* homocysteine, *HEI* healthy eating index, *N* sample size, *SEM* standard error of the mean.

^a^Depressive symptoms scores include the CES-D total score, the CES-D domain 1 score [depressive affect], the CES-D domain 2 score [interpersonal problems], the CES-D domain 3 score [somatic complaints] and the CES-D domain 4 score [positive affect].

**Fig. 4:**
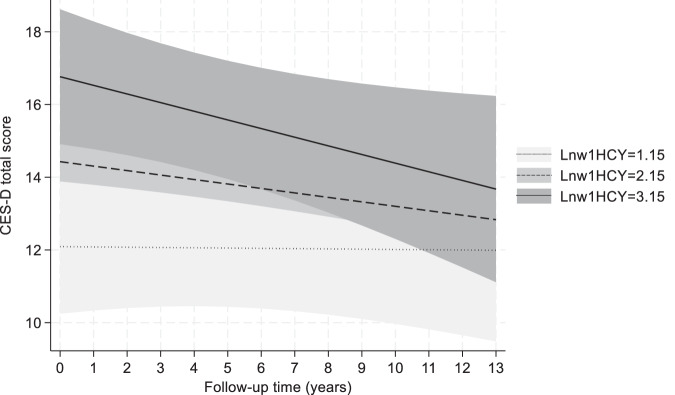
Predictive margins of CES-D total score from Model 1 of LnHcy vs. CES-D total score in the overall sample – HANDLS (2004–2017). Notes: CES-D Center for Epidemiologic Studies-Depression, HANDLS healthy aging in neighborhoods of diversity across the lifespan study. Based on Model 1 mixed-effects linear regression model with outcome being CES-D total score and main exposure LnHcy at baseline visit centered at 2.15. Model adjusted for baseline age, sex, race, poverty status and inverse mills ratio. Three trajectories are shown for LnHcy levels of 1.15, 2.15 and 3.15 to illustrate difference in baseline CES-D total score per unit increase in the exposure (β ± SE: +2.337 ± 0.902, *P* = 0.010).

The associations of LnHcy_v1_ with depressive symptoms over time (total and domain-specific scores), both baseline and annualized change are presented in Table 2, overall, and across sex and race. Overall, and after adjusting for multiple testing, a positive cross-sectional relationship was observed baseline LnHcy and CES-D total scores in Model 1 (β (standard error [SE]) = + 2.337 (0.902), *P* = 0.010), a relationship that was slightly attenuated in Model 2 but remained statistically significant [Model 2 (β (SE) = 1.825 (0.883), *P* = 0.039). The finding from Model 1 is illustrated in Fig. 4 as predictive margins of CES-D total scores across time based on this model. Among women, LnHcy_v1_ was only associated with reduced scores on the positive affect sub-domain (Domain #4) in both the reduced (Model 1: *β* (SE) = −0.86 (0.30), *P* = 0.004) and the fully adjusted models (Model 2: *β* (SE) = −0.68 (0.30), *P* = 0.025). Importantly, when we stratified the analyses by levels of anxiety (below vs. above median), heterogeneity across these groups were detected. Most notably, among individuals below median anxiety, LnHcy was associated with a faster increase in CES-D domain 2 score over time (“interpersonal problems”: *β* (SE) = 0.043 (0.018), *P* = 0.018) for Model 1; and *β* (SE) = 0.041 (0.018), *P* = 0.024) for Model 2). In contrast, among individuals above median anxiety, LnHcy showed a strong positive cross-sectional association with CES-D total score (*β* (SE) = 4.02 (1.46), *P* = 0.006) for Model 1; and *β* (SE) = 3.59 (1.45), *P* = 0.013) for Model 2).

**Table 2:** Parameter estimates from mixed effects linear regression models that regressed depressive symptoms total and domain-specific scores (baseline and between-visit change) on Log_e_-transformed homocysteine (HCY) values.

	X = Ln(Homocysteine) at v1 (LnHcy)			
	Model 1^a^		Model 2^b^	
Overall^c^ (*N* = 1460, K = 2.8):	β (SE)	*P* value	β (SE)	*P* value
CES-D total score:				
LnHcy	+2.337 (0.902)	0.010	+1.825 (0.883)	0.039
LnHcy × Time	−0.115 (0.108)	0.286	−0.114 (0.109)	0.294
CES-D domain 1 score:				
LnHcy	+0.951 (0.394)	0.016	+0.773 (0.389)	0.047
LnHcy × Time	−0.067 (0.047)	0.156	−0.066 (0.048)	0.166
CES-D domain 2 score:				
LnHcy	+0.085 (0.106)	0.422	+0.051 (0.105)	0.626
LnHcy × Time	−0.003 (0.015)	0.839	−0.002 (0.015)	0.895
CES-D domain 3 score:				
LnHcy	+0.784 (0.356)	0.028	+0.570 (0.348)	0.102
LnHcy × Time	−0.010 (0.047)	0.831	−0.014 (0.048)	0.770
CES-D domain 4 score:				
LnHcy	−0.481 (0.210)	0.022	−0.381 (0.208)	0.067
LnHcy × Time	+0.036 (0.032)	0.265	+0.033 (0.032)	0.302
MEN (*n* = 619, K = 2.7)**:**				
CES-D total score:				
LnHcy	+1.587 (1.195)	0.184	+1.401 (1.170)	0.231
LnHcy × Time	−0.191 (0.148)	0.198	−0.182 (0.148)	0.220
CES-D domain 1 score:				
LnHcy	+1.026 (0.506)	0.043	+0.943 (0.482)	0.050
LnHcy × Time	−0.142 (0.062)	0.021	−0.132 (0.062)	0.033
CES-D domain 2 score:				
LnHcy	+0.033 (0.162)	0.839	+0.010 (0.160)	0.948
LnHcy × Time	−0.011 (0.022)	0.636	−0.010 (0.023)	0.672
CES-D domain 3 score:				
LnHcy	+0.536 (0.485)	0.269	+0.472 (0.475)	0.321
LnHcy × Time	−0.038 (0.067)	0.568	−0.036 (0.067)	0.592
CES-D domain 4 score:				
LnHcy	−0.040 (0.285)	0.890	−0.001 (0.283)	0.998
LnHcy × Time	+0.017 (0.046)	0.719	+0.016 (0.046)	0.732
WOMEN (*n* = 841, K = 2.8)**:**				
CES-D total score:				
LnHcy	+2.932 (1.325)	0.027	+1.818 (1.300)	0.162
LnHcy × Time	+0.003 (0.156)	0.986	+0.003 (0.159)	0.985
*CES-D domain 1 score:*				
LnHcy	+0.925 (0.586)	0.114	+0.480 (0.559)	0.390
LnHcy × Time	+0.001 (0.069)	0.984	+0.004 (0.071)	0.957
*CES-D domain 2 score:*				
LnHcy	+0.163 (0.141)	0.246	+0.099 (0.142)	0.486
LnHcy × Time	+0.003 (0.020)	0.884	+0.006 (0.020)	0.775
CES-D domain 3 score:				
LnHcy	+0.892 (0.513)	0.082	+0.446 (0.501)	0.373
LnHcy × Time	+0.037 (0.066)	0.574	+0.029 (0.067)	0.666
CES-D domain 4 score:				
LnHcy	−0.863 (0.303)	0.004	−0.680 (0.303)	0.025
LnHcy × Time	+0.040 (0.045)	0.369	+0.036 (0.046)	0.430
WHITE (*N* = 630, K = 2.8)**:**				
CES-D total score:				
LnHcy	+1.807 (1.479)	0.222	+1.362 (1.458)	0.350
LnHcy × Time	−0.205 (0.186)	0.270	−0.229 (0.189)	0.225
CES-D domain 1 score:				
LnHcy	+0.535 (0.616)	0.384	+0.403 (0.613)	0.511
LnHcy × Time	−0.094 (0.081)	0.249	−0.103 (0.082)	0.209
CES-D domain 2 score:				
LnHcy	+0.087 (0.165)	0.600	+0.055 (0.165)	0.741
LnHcy × Time	−0.005 (0.024)	0.849	−0.003 (0.024)	0.915
CES-D domain 3 score:				
LnHcy	+0.599 (0.567)	0.291	+0.407 (0.554)	0.462
LnHcy × Time	−0.055 (0.077)	0.469	−0.070 (0.078)	0.369
CES-D domain 4 score:				
LnHcy	−0.487 (0.353)	0.167	−0.385 (0.351)	0.272
LnHcy × Time	+0.049 (0.053)	0.352	+0.052 (0.054)	0.336
**African American** (*n* = 830, K = 2.7)**:**				
CES-D total score:				
LnHcy	+2.593 (1.150)	0.024	+2.064 (1.114)	0.064
LnHcy × Time	−0.030 (0.135)	0.827	−0.023 (0.136)	0.868
CES-D domain 1 score:				
LnHcy	+1.224 (0.504)	0.015	+1.013 (0.473)	0.032
LnHcy × Time	−0.046 (0.058)	0.431	−0.039 (0.058)	0.503
CES-D domain 2 score:				
LnHcy	+0.110 (0.139)	0.428	+0.070 (0.138)	0.610
LnHcy × Time	−0.004 (0.019)	0.839	−0.003 (0.019)	0.880
CES-D domain 3 score:				
LnHcy	+0.821 (0.458)	0.073	+0.612 (0.444)	0.168
LnHcy × Time	+0.024 (0.060)	0.691	+0.023 (0.061)	0.706
CES-D domain 4 score:				
LnHcy	−0.447 (0.259)	0.085	−0.353 (0.257)	0.169
LnHcy × Time	+0.014 (0.041)	0.724	+0.009 (0.041)	0.820
**Below median anxiety score** (*n* = 610, K = 2.8)**:**				
CES-D total score:				
LnHcy	+1.288 (1.022)	0.208	+0.918 (1.008)	0.362
LnHcy × Time	+0.055 (0.141)	0.695	+0.038 (0.142)	0.789
CES-D domain 1 score:				
LnHcy	+0.052 (0.398)	0.895	−0.024 (0.422)	0.954
LnHcy × Time	+0.029 (0.060)	0.631	+0.011 (0.059)	0.848
CES-D domain 2 score:				
LnHcy	−0.039 (0.121)	0.748	−0.042 (0.122)	0.731
LnHcy × Time	+0.043 (0.018)	0.018	+0.041 (0.018)	0.024
CES-D domain 3 score:				
LnHcy	+0.817 (0.444)	0.066	+0.599 (0.450)	0.183
LnHcy × Time	+0.021 (0.064)	0.741	+0.018 (0.064)	0.782
CES-D domain 4 score:				
LnHcy	−0.390 (0.251)	0.120	−0.323 (0.251)	0.198
LnHcy × Time	+0.040 (0.044)	0.361	+0.040 (0.044)	0.357
**Above median anxiety score** (*n* = 571, K = 2.8)**:**				
CES-D total score:				
LnHcy	+4.015 (1.461)	0.006	+3.587 (1.451)	0.013
LnHcy × Time	−0.178 (0.194)	0.361	−0.195 (0.197)	0.321
CES-D domain 1 score:				
LnHcy	+1.834 (0.666)	0.006	+1.679 (0.647)	0.009
LnHcy × Time	−0.095 (0.085)	0.264	−0.099 (0.086)	0.248
CES-D domain 2 score:				
LnHcy	+0.400 (0.183)^d^	0.029	+0.362 (0.183)^d^	0.047
LnHcy × Time	−0.044 (0.027)^d^	0.101	−0.043 (0.027)^d^	0.11
CES-D domain 3 score:				
LnHcy	+1.120 (0.549)	0.041	+0.927 (0.543)	0.088
LnHcy × Time	−0.029 (0.083)	0.729	−0.039 (0.083)	0.638
CES-D domain 4 score:				
LnHcy	−0.694 (0.352)	0.049	−0.628 (0.362)	0.082
LnHcy × Time	+0.012 (0.054)	0.828	+0.015 (0.056)	0.791

Results are presented for models fit to the overall sample of HANDLS 2004–2017 participants (*N* = 1460) and by stratifying variables.

*CES-D* Center for Epidemiological Studies Depression, *Hcy* Homocysteine, *K* Mean number of visits per subject, *N* Sample size, *SE* Standard error.

^a^Model 1 is adjusted for age, sex, race, poverty status, inverse mills ratio as well as time on study between visits 1 and 2 (in years) and its interaction with homocysteine and covariates.

^b^Model 2 is adjusted for age, sex, race, poverty status, education, smoking, drug use, 2010 healthy eating index, body mass index, inverse mills ratio as well as time on study between visits 1 and 2 (in years) and its interaction with homocysteine and covariates.

^c^Depressive symptoms scores include the CES-D total score, the CES-D domain 1 score [depressive affect], the CES-D domain 2 score [interpersonal problems], the CES-D domain 3 score [somatic complaints] and the CES-D domain 4 score [positive affect].

^d^*P* < 0.05 for null hypothesis that LnHcy × Z = 0 or LnHcy × Time × Z = 0 in unstratified model with Z being sex, race or anxiety status.

The probability of belonging to the “High” LnHcy trajectory was *z*-scored (Hcyt_raj_) and entered into another set of mixed-effects linear regression models with outcome being CES-D total and sub-domain scores (baseline and between-visit change), overall, and by stratifying variables (Table 3). Overall, a “High” LnHcy trajectory probability (*z*-scored), or Hcy_traj_, was directly associated with baseline CES-D total score for Model 1 (β (SE) = 0.74 (0.29), *P* = 0.009), an association that was slightly attenuated in Model 2 (β (SE) = 0.64 (0.28), *P* = 0.021). This association was mainly driven by the relationship between Hcy_traj_ and the interpersonal problems (Domain 2 score) and somatic complaints (Domain 3 score) sub-domains. Hcy_traj_ was also linked to a higher score on the depressed affect sub-domain (Domain 1 score) among men at baseline, an association that remained statistically significant in Model 2. Among individuals with an above median anxiety score, Hcy_traj_ was inversely related to the annualized rate of increase in the “interpersonal problems” sub-domain (Domain 2 score) in the reduced and fully adjusted models.

**Table 3:** Parameter estimates from mixed effects linear regression models that regressed depressive symptoms total and domain-specific scores (baseline and between-visit change) on homocysteine trajectory (Hcy_traj_).

	Hcy_traj_: probability of “High” LnHcy, *z*-scored			
	Model 1^a^		Model 2^b^	
	β (SE)	*P*	β (SE)	*P*
**Overall**^**c**^ (*N* = 1457, K = 2.8):				
CES-D total score:				
Hcy _traj_	+0.742 (0.285)	0.009	+0.640 (0.278)	0.021
Hcy _traj_ × Time	−0.021 (0.034)	0.535	−0.022 (0.034)	0.518
CES-D domain 1 score:				
Hcy _traj_	+0.258 (0.125)	0.039	+0.224 (0.123)	0.068
Hcy _traj_ × Time	−0.010 (0.015)	0.492	−0.010 (0.015)	0.504
CES-D domain 2 score:				
Hcy _traj_	+0.075 (0.033)	0.025	+0.069 (0.033)	0.038
Hcy _traj_ × Time	−0.006 (0.005)	0.161	−0.006 (0.005)	0.164
CES-D domain 3 score:				
Hcy _traj_	+0.311 (0.112)	0.006	+0.265 (0.110)	0.016
Hcy _traj_ × Time	−0.001 (0.015)	0.948	−0.001 (0.015)	0.924
CES-D domain 4 score:				
Hcy _traj_	−0.077 (0.066)	0.246	−0.059 (0.066)	0.371
Hcy _traj_ × Time	+0.001 (0.010)	0.900	+0.002 (0.010)	0.848
**Men** (*n* = 618, K = 2.7):				
CES-D total score:				
Hcy _traj_	+0.773 (0.340)	0.023	+0.698 (0.335)	0.037
Hcy _traj_ × Time	−0.003 (0.041)	0.939	−0.001 (0.042)	0.980
CES-D domain 1 score:				
Hcy _traj_	+0.397 (0.144)	0.006	+0.370 (0.144)	0.010
Hcy _traj_ × Time	−0.016 (0.017)	0.360	−0.014 (0.017)	0.405
CES-D domain 2 score:				
Hcy _traj_	+0.086 (0.046)	0.062	+0.077 (0.046)	0.096
Hcy _traj_ × Time	−0.007 (0.006)	0.281	−0.006 (0.006)	0.302
CES-D domain 3 score:				
Hcy _traj_	+0.272 (0.138)	0.049	+0.248 (0.136)	0.070
Hcy _traj_ × Time	+0.004 (0.019)	0.825	+0.005 (0.019)	0.793
CES-D domain 4 score:				
Hcy _traj_	−0.025 (0.082)	0.755	−0.007 (0.081)	0.935
Hcy _traj_ × Time	−0.013 (0.013)	0.295	−0.014 (0.013)	0.289
**Women** (*n* = 839, K = 2.8):				
CES-D total score:				
Hcy _traj_	+0.673 (0.469)	0.151	+0.409 (0.456)	0.370
Hcy _traj_ × Time	−0.021 (0.055)	0.698	−0.021 (0.056)	0.703
CES-D domain 1 score:				
Hcy _traj_	+0.085 (0.207)	0.682	−0.020 (0.203)	0.921
Hcy _traj_ × Time	+0.001 (0.025)	0.971	+0.001 (0.025)	0.974
CES-D domain 2 score:				
Hcy _traj_	+0.074 (0.050)	0.139	+0.060 (0.050)	0.227
Hcy _traj_ × Time	−0.006 (0.007)	0.366	−0.006 (0.007)	0.392
CES-D domain 3 score:				
Hcy _traj_	+0.319 (0.181)	0.078	+0.213 (0.176)	0.225
Hcy _traj_ × Time	+0.002 (0.023)	0.944	+0.001 (0.024)	0.953
CES-D domain 4 score:				
Hcy _traj_	−0.147 (0.108)	0.171	−0.102 (0.107)	0.339
Hcy _traj_ × Time	+0.016 (0.016)	0.323	+0.015 (0.016)	0.349
**White** (*n* = 627, K = 2.8)**:**				
CES-D total score:				
Hcy _traj_	+0.736 (0.493)	0.136	+0.660 (0.489)	0.177
Hcy _traj_ × Time	+0.051 (0.061)	0.402	+0.042 (0.062)	0.495
CES-D domain 1 score:				
Hcy _traj_	+0.261 (0.206)	0.207	+0.231 (0.213)	0.278
Hcy _traj_ × Time	+0.009 (0.027)	0.745	+0.008 (0.027)	0.775
CES-D domain 2 score:				
Hcy _traj_	+0.091 (0.055)	0.101	+0.085 (0.055)	0.125
Hcy _traj_ × Time	+0.006 (0.008)	0.430	+0.006 (0.008)	0.421
CES-D domain 3 score:				
Hcy _traj_	+0.296 (0.190)	0.119	+0.263 (0.185)	0.155
Hcy _traj_ × Time	+0.020 (0.025)	0.419	+0.016 (0.026)	0.539
CES-D domain 4 score:				
Hcy _traj_	−0.022 (0.119)	0.856	−0.006 (0.118)	0.959
Hcy _traj_ × Time	−0.021 (0.017)	0.227	−0.019 (0.018)	0.272
**African American** (*n* = 830, K = 2.7)**:**				
CES-D total score:				
Hcy _traj_	+0.676 (0.352)	0.054	+0.557 (0.341)	0.102
Hcy _traj_ × Time	−0.038 (0.041)	0.357	−0.036 (0.041)	0.378
CES-D domain 1 score:				
Hcy _traj_	+0.241 (0.154)	0.119	+0.196 (0.145)	0.176
Hcy _traj_ × Time	−0.015 (0.018)	0.382	−0.014 (0.018)	0.431
CES-D domain 2 score:				
Hcy _traj_	+0.069 (0.042)	0.102	+0.060 (0.042)	0.155
Hcy _traj_ × Time	−0.013 (0.006)	0.029	−0.012 (0.006)	0.033
CES-D domain 3 score:				
Hcy _traj_	+0.278 (0.140)	0.047	+0.229 (0.136)	0.093
Hcy _traj_ × Time	−0.005 (0.018)	0.796	−0.004 (0.018)	0.811
CES-D domain 4 score:				
Hcy _traj_	−0.095 (0.079)	0.233	+0.071 (0.079)	0.368
Hcy _traj_ × Time	+0.007 (0.012)	0.553	+0.007 (0.012)	0.588
**Below median anxiety score** (*n* = 608, K = 2.8)**:**				
CES-D total score:				
Hcy _traj_	+0.412 (0.309)	0.182	+0.343 (0.322)	0.286
Hcy _traj_ × Time	+0.005 (0.045)	0.912	−0.007 (0.045)	0.880
CES-D domain 1 score:				
Hcy _traj_	+0.047 (0.128)	0.714	+0.032 (0.126)	0.802
Hcy _traj_ × Time	+0.008 (0.019)	0.687	+0.005 (0.019)	0.783
CES-D domain 2 score:				
Hcy _traj_	+0.039 (0.039)	0.316	+0.039 (0.039)	0.309
Hcy _traj_ × Time	+0.009 (0.006)	0.126	+0.008 (0.006)	0.152
CES-D domain 3 score:				
Hcy _traj_	+0.277 (0.142)	0.052	+0.226 (0.140)	0.106
Hcy _traj_ × Time	−0.003 (0.020)	0.872	-0.005 (0.020)	0.818
CES-D domain 4 score:				
Hcy _traj_	−0.013 (0.081)	0.872	−0.002 (0.084)	0.979
Hcy _traj_ × Time	+0.011 (0.014)	0.431	+0.014 (0.015)	0.338
**Above median anxiety score** (*n* = 570, K = 2.8)**:**				
CES-D total score:				
Hcy _traj_	+0.293 (0.451)	0.516	+0.285 (0.446)	0.522
Hcy _traj_ × Time	−0.017 (0.059)	0.774	−0.021 (0.059)	0.718
CES-D domain 1 score:				
Hcy _traj_	+0.074 (0.206)	0.720	+0.073 (0.198)	0.712
Hcy _traj_ × Time	−0.013 (0.026)	0.619	−0.015 (0.026)	0.567
CES-D domain 2 score:				
Hcy _traj_	+0.096 (0.056)	0.088	+0.093 (0.056)	0.096
Hcy _traj_ × Time	−0.023 (0.008)^d^	0.005	−0.022 (0.008)^d^	0.006
CES-D domain 3 score:				
Hcy _traj_	+0.157 (0.169)	0.353	+0.154 (0.166)	0.353
Hcy _traj_ × Time	+0.000 (0.025)	0.994	−0.002 (0.025)	0.922
CES-D domain 4 score:				
Hcy _traj_	+0.040 (0.109)	0.716	+0.044 (0.111)	0.694
Hcy _traj_ × Time	−0.020 (0.016)	0.209	−0.020 (0.017)	0.231

Results are presented for models fit to the overall sample of HANDLS 2004–2017 participants (*N* = 1460) and by stratifying variables.

*CES-D* Center for Epidemiological Studies Depression, *Hcy*_*traj*_ z-transformed probability of belonging to a group with higher homocysteine over time according to group-based trajectory modeling, *K* mean number of visits per subject, *N* sample size, *SE* standard error.

^a^Model 1 is adjusted for age, sex, race, poverty status, inverse mills ratio as well as time on study in years between visits 1 and 3 and its interaction with homocysteine trajectory and covariates.

^b^Model 2 is adjusted for age, sex, race, poverty status, education, literacy, smoking, drug use, 2010 healthy eating index, body mass index, inverse mills ratio as well as time on study in years between visits 1 and 3 and its interaction with homocysteine trajectory and covariates.

^c^Depressive symptoms scores include the CES-D total score, the CES-D domain 1 score [depressive affect], the CES-D domain 2 score [interpersonal problems], the CES-D domain 3 score [somatic complaints] and the CES-D domain 4 score [positive affect].

^d^*P* < 0.05 for null hypothesis that LnHcy × Z = 0 or LnHcy × Time × Z = 0 in unstratified model with Z being sex, race or anxiety status.

Four sensitivity analyses were also conducted. In the first sensitivity analysis, LnHcy at visit 1 was studied in relation to the Log_e_ odds of high anxiety (Table S1). No association was detected in both the reduced and fully adjusted models. In a second sensitivity analysis (Table S2), annualized rate of change in Hcy between visits 1 and 2 was studied in relation to CES-D total and sub-scores in the overall sample. After adjustment for multiple testing, annualized change in LnHcy between the first two visits was not associated with neither baseline nor annualized change in CES-D scores, in both reduced and fully adjusted models. Third, a mixed-effects linear regression model with LnHcy as the outcome was conducted in the overall sample examining its association with various exposures, including baseline CES-D total score, ordinal anxiety score, probability of higher anxiety score and anxiety disorder at baseline (Table S3). Most notably, baseline CES-D total score was associated with LnHcy_v1_ in both the reduced and fully adjusted models. In contrast, a higher probability of elevated anxiety over time was associated with faster increase in LnHcy over time, a finding only detected in the reduced model. In the fourth sensitivity analysis (Table S4), the two main Hcy exposures of interest were tested as predictors for incidence of EDS over time, using a series of fully adjusted Cox proportional hazards models, while stratifying and testing for potential effect modification by sex, race, poverty status and anxiety score levels. Baseline Hcy was not associated with incident EDS overall. In fact, there was strong heterogeneity in this association across race, poverty status and anxiety levels. Most notably, baseline Hcy was inversely associated with incident EDS among White participants and participants living above poverty while being positively associated with this outcome among African American adults, adults with low level of anxiety at baseline, and adults living below poverty (*P* < 0.05 for 2-way interaction terms between baseline Hcy exposure and those 3 effect modifiers). In contrast, Hcy_traj_ was positively associated with incident EDS overall (HR = 1.09, 95% CI: 1.03–1.14, *P* = 0.001), more strongly so among women and individuals living below poverty (*P* < 0.05 for 2-way interaction between Hcy_traj_ and those 2 effect modifiers). For Hcy_traj_, the strongest association with incident EDS was noted in the “Below poverty” stratum: HR = 1.22, 95% CI: 1.13–1.18, *P* < 0.001.

## Discussion

In this prospective cohort study of 1460 urban adults, 30 to 64 years of age at baseline, mean LnHcy_v1_ and CES-D total scores were estimated at 2.15 and 14.01, respectively. Overall, a positive cross-sectional relationship was observed LnHcy_v1_ and CES-D total scores in Model 1 (β ([SE]) = + 2.337 (0.902), *P* = 0.010), a relationship that was slightly attenuated in Model 2 [(β (SE) = + 1.825 (0.883), *P* = 0.039). However, heterogeneity was detected across anxiety levels, with individuals below median anxiety experiencing faster increases in CES-D domain 2 scores with increased LnHcy_v1_ (interpersonal problems: *β* [SE] = 0.041 [0.018], *P* = 0.024). A “High” LnHcy trajectory probability (*z*-scored), or Hcy_traj_, was directly associated with baseline CES-D total score for Model 1 (β (SE) = + 0.74 (0.29), *P* = 0.009), an association that was slightly attenuated in Model 2 (β (SE) = + 0.64 (0.28), *P* = 0.021). This association was mainly driven by the relationship between Hcy_traj_ and the interpersonal problems and somatic complaints sub-domains. Among individuals with an above median anxiety score, Hcy_traj_ was inversely related to the annualized rate of increase in the “interpersonal problems” sub-domain (Domain 2 score) in the reduced and fully adjusted models. Hcy_traj_ was positively associated with incident elevated depressive symptoms (CES-D total score ≥ 16) overall (fully adjusted model: HR = 1.09, 95% CI: 1.03–1.14, *P* = 0.001), though more strongly so among women and individuals living below poverty.

Though research in this area of work is limited, prior studies suggested that higher levels of HCY were related to depressive episodes, symptoms, and risk for depression and anxiety disorders, though it remains unclear if anxiety partially explains these relationships [13, 17–22]. Differences in sample compositions across age, sex, nationality, and prior medical history of psychiatric disorders may be reasons for these discrepancies. Furthermore, studies have varied on their assessments of depression, depressive symptoms, and anxiety, as well as that of other conditions such as PTSD. The growing body of work in this area warrants further inquiry into how these relationships develop over time and to what extent the heterogeneity across anxiety levels observed in the present study highlight more nuanced influences per depression- or anxiety-related disorders.

The cross-sectional and longitudinal evidence of the positive association of HCY and symptoms of depression are consistent with the findings from other studies [15, 16]. Most notably, a systematic review and meta-analysis comparing homocysteine levels in healthy subjects and those with depression found that individuals with higher homocysteine levels had a higher risk of depression [15]. This was found in studies using various depression diagnostic tools, including DSM-IV, GDS, ZDRS, and BDI-II [15]. The review and meta-analysis also found that participants with hyperhomocysteinaemia had a higher chance of depression [15]. The findings suggest that future research should focus on the tools used for depression assessment to better understand the relationship between homocysteine levels and depression [15]. A cross-sectional study along with a systematic review, and meta-analysis were conducted on 3752 men aged 70 years or older [16]. The results showed that the odds ratio of prevalent depression increased 4% with every unit increase of total homocysteine (tHcy) [16]. The methylenetetrahydrofolate reductase (MTHFR) C677T TT genotype was 0.19 mg/L higher among participants with the MTHFR C677T TT genotype compared to the CC genotype [16]. The meta-analysis showed that older adults with high tHcy had an increased risk of depression, and TT carriers were 22% more likely than CC carriers to have current depression or a history of depression [16]. The triangular association between the MTHFR genotype, tHcy, and depression implies that higher concentrations of tHcy increase the risk of depression [16]. Confirmatory data from sufficiently powered randomized trials of homocysteine-lowering therapy are now required to test the causal relationship between tHcy and depression [16].

Nevertheless, a study conducted in the Longitudinal Aging Study Amsterdam (LASA) involving 1352 men and women aged ⩾65 years, assessed depressive symptoms six times from 1995/1996 to 2011/2012 [14]. Multiple linear regression and mixed models were used to assess the associations of vitamin B-12 as well as Hcy with depressive symptoms over 16 years [14]. The results showed that vitamin B-12 was not cross-sectionally or prospectively associated with depressive symptoms, and no association was found with incident depression [14]. For Hcy, no associations were found, except for a lower risk of depression in younger participants [14]. The study concluded that further research is needed to understand the influence of Hcy metabolism on mental health [14]. Our present study detected a similar inverse association between baseline Hcy and incident EDS among White adults and individuals living over poverty with no detectable association overall. In contrast, there was a positive association detected among African American adults and individuals living below poverty. Therefore, the inverse association detected in the previous study may be specific to middle-aged adults of higher SES, independently of other socio-demographic, lifestyle and health-related factors.

Although the relationship of HCY to depression was first noted in 1970 [9], the exact mechanisms explaining the association remain unclear. High levels of homocysteine can result from a variety of reasons including a dietary deficiency of vitamins B_6,_ B_12,_ and folate, genetic variation of the enzymes essential for the metabolism of homocysteine such as MTHFR and cystathionine beta-synthase, enzymes, gastric atrophy, inflammatory bowel disease and methionine loading [9, 16]. Elevated HCY may result in deficiency of methyl transfer reactions, resulting in deficits to neurotransmitter synthesis, and is associated with reduced cortical and hippocampal volumes in the brain. These changes may explain how HCY levels mediate symptoms of depression [44]. The effects of HCY on depression have been reported to be independent of dementia [45]. However, the interaction of HCY and depression can influence cognitive performance [46]. Evaluated HCY and depression appear to be associated with white matter damage, gray matter atrophy, β-amyloid deposition, and cardiovascular disease risk [46, 47]. Yet not all studies have found the association between HCY and depression which could reflect the many causes for elevated HCY, including genetic predisposition and brain disease, and the several types of depression in samples of different ethnicities and ages [9, 48].

A growing interest in Plasma HCY and its sequelae has spurred a set of evidence linking HCY to adverse outcomes for brain health. Much of this interest has grown out of observational studies linking high plasma HCY and allelic variants of the MTHFR gene to psychiatric abnormalities [9, 49]. HCY production is controlled through one-carbon metabolism [50, 51]. Methionine synthase, a B12-dependent enzyme, is a key enzyme that governs the utilization of HCY [50, 51]. Vitamin B12 deficiency or a mutation in the enzyme slow the conversion of HCY to methionine, leading to the accumulation of HCY [50, 51]. An inborn error of metabolism in the MTHFR gene, which is responsible for the production of the other substrate of the methionine synthase reaction, 5-methyl THF, can also cause HCY accumulation [52]. Animal studies have shown that loss-of-function mutations in MTHFR decrease levels of monoamine neurotransmitters in key regions of the brain, such as glutamate in the amygdala and γ-aminobutyric acid in the thalamus [53]. Hyperhomocysteinemia has also been linked to the production of homocysteic acid, a compound that is known to be neurotoxic to dopaminergic regions of the brain and that functions as an N-methyl-D-aspartate (NMDA) receptor agonist [54]. Indeed, it appears that the downstream neurotoxic effects of HCY on dopaminergic circuits and the changes in monoamine neurotransmitter concentrations may help explain the observed associations from our study, which we find are also consistent across several other epidemiological studies [9, 55].

Our study has several strengths including its being the first study conducted among ethnically and socio-economically diverse urban middle-aged adults to examine these intricate research questions. Second, the study was well-powered to examine associations across sex and race, as well as across anxiety levels. Furthermore, analyses included advanced techniques such as multiple imputation coupled with mixed-effects linear regression models and 2-stage Heckman selection. There was also a wealth of data available to control for potential confounders in all our models. A few limitations are worth considering when interpreting study results. First, subsamples of the initial HANDLS participants were used to assess potential associations between HCY and depressive symptoms, which may have resulted in selection bias. Second, as a large number of exposure, outcome, and covariate assessment factors were self-reported, measurement errors are probably present and could result in skewed measures of association. Third, our ability to compare our findings with published literature may have been hampered by various criteria used to categorize HCY as well as different means of measuring it in serum. Fourth, there’s a chance that the amount of time spent in follow-up between HANDLS Visits 1, 2, and 3 was insufficient to see any clinically significant improvements in depression symptoms. Similarly, fluctuation of depressive symptoms over time cannot be fully captured by the total CES-D score. Future research should therefore investigate proposed correlations over extended follow-up periods. Fifth, even after adjusting for a number of factors, residual confounding is likely because HANDLS is an observational study, which means we were unable to demonstrate causal associations. Sixth, while analyzing HCY in connection to depressive symptoms, the impact of interaction effects by sex and race might have been understated. Lastly, the HANDLS study’s sampling design limits the generalizability of the study’s findings to middle-aged and older persons living in metropolitan areas across the United States.

## Conclusion

Among urban middle-aged adults, baseline and “high trajectory” of LnHcy were positively associated with depressive symptoms and elevated depressive symptom incidence, in a sex-, race-, poverty status- and anxiety-level specific manner. More research is needed using longitudinal designs with larger sample sizes and longer follow-up times, in order to replicate and confirm our present study findings.

## Supplementary information

Online Supplementary Materials

## Data Availability

The study protocol (09-AG-N248) received approval from the National Institute on Environmental Health Sciences’ Institutional Review Board (IRB) of the National Institutes of Health (NIH). Upon request, data can be made available to researchers with approved proposals, after they have agreed to confidentiality as required by our IRB. Policies are publicized on: https://handls.nih.gov. Data access request can be sent to principal investigators (PI) or the study manager, Jennifer Norbeck at norbeckje@mail.nih.gov. These data are owned by the National Institute on Aging at the NIH. The PIs have made those data restricted to the public for two main reasons: “(1) The study collects medical, psychological, cognitive, and psychosocial information on racial and poverty differences that could be misconstrued or willfully manipulated to promote racial discrimination; and (2) Although the sample is fairly large, there are sufficient identifiers that the PIs cannot guarantee absolute confidentiality for every participant as we have stated in acquiring our confidentiality certificate.” [56]

## References

[1] Hu T, Zhao X, Wu M, Li Z, Luo L, Yang C, et al. Prevalence of depression in older adults: a systematic review and meta-analysis. Psychiatry Res. 2022;311:114511. 35316691 10.1016/j.psychres.2022.114511

[2] Zenebe Y, Akele B, Selassie MW, Necho M. Prevalence and determinants of depression among old age: a systematic review and meta-analysis. Ann Gen Psychiatry. 2021;20:55. 34922595 10.1186/s12991-021-00375-x PMC8684627

[3] Obuobi-Donkor G, Nkire N, Agyapong VIO. Prevalence of major depressive disorder and correlates of thoughts of death, suicidal behaviour, and death by suicide in the geriatric population—a general review of literature. Behav Sci. 2021;11:142. 34821603 10.3390/bs11110142 PMC8614881

[4] Hohls JK, Konig HH, Quirke E, Hajek A. Anxiety, depression and quality of life—a systematic review of evidence from longitudinal observational studies. Int J Environ Res Public Health. 2021;18:12022. 34831779 10.3390/ijerph182212022 PMC8621394

[5] Wei J, Hou R, Zhang X, Xu H, Xie L, Chandrasekar EK, et al. The association of late-life depression with all-cause and cardiovascular mortality among community-dwelling older adults: systematic review and meta-analysis. Br J Psychiatry. 2019;215:449–55. 30968781 10.1192/bjp.2019.74

[6] Krause N. Life stress as a correlate of depression among older adults. Psychiatry Res. 1986;18:227–37. 3749384 10.1016/0165-1781(86)90110-1

[7] Rauch SA, Morales KH, Zubritsky C, Knott K, Oslin D. Posttraumatic stress, depression, and health among older adults in primary care. Am J Geriatr Psychiatry. 2006;14:316–24. 16582040 10.1097/01.JGP.0000199382.96115.86

[8] Zannas AS, McQuoid DR, Payne ME, Steffens DC, MacFall JR, Ashley-Koch A, et al. Negative life stress and longitudinal hippocampal volume changes in older adults with and without depression. J Psychiatr Res. 2013;47:829–34. 23478048 10.1016/j.jpsychires.2013.02.008 PMC3622849

[9] Folstein M, Liu T, Peter I, Buel J, Arsenault L, Scott T, et al. The homocysteine hypothesis of depression. Am J Psychiatry. 2007;164:861–7. 17541043 10.1176/ajp.2007.164.6.861

[10] Bottiglieri T, Laundy M, Crellin R, Toone BK, Carney MW, Reynolds EH. Homocysteine, folate, methylation, and monoamine metabolism in depression. J Neurol Neurosurg Psychiatry. 2000;69:228–32. 10896698 10.1136/jnnp.69.2.228 PMC1737050

[11] Ganguly P, Alam SF. Role of homocysteine in the development of cardiovascular disease. Nutr J. 2015;14:1–10. 25577237 10.1186/1475-2891-14-6 PMC4326479

[12] Marinou K, Antoniades C, Tousoulis D, Pitsavos C, Goumas G, Stefanadis C. Homocysteine: a risk factor for coronary artery. Hellenic J Cardiol. 2005;46:59–67. 15807397

[13] Levine J, Timinsky I, Vishne T, Dwolatzky T, Roitman S, Kaplan Z, et al. Elevated serum homocysteine levels in male patients with PTSD. Depress Anxiety. 2008;25:E154–E157. 17994587 10.1002/da.20400

[14] Elstgeest LE, Brouwer IA, Penninx BW, van Schoor NM, Visser M. Vitamin B(12), homocysteine and depressive symptoms: a longitudinal study among older adults. Eur J Clin Nutr. 2017;71:468–75. 28145420 10.1038/ejcn.2016.224

[15] Moradi F, Lotfi K, Armin M, Clark CCT, Askari G, Rouhani MH. The association between serum homocysteine and depression: a systematic review and meta-analysis of observational studies. Eur J Clin Invest. 2021;51. 10.1111/eci.13486. 10.1111/eci.13486 33423269

[16] Almeida OP, McCaul K, Hankey GJ, Norman P, Jamrozik K, Flicker L. Homocysteine and depression in later life. Arch Gen Psychiatry. 2008;65:1286–94. 18981340 10.1001/archpsyc.65.11.1286

[17] de Vries G-J, Lok A, Mocking R, Assies J, Schene A, Olff M. Altered one-carbon metabolism in posttraumatic stress disorder. J Affect Disord. 2015;184:277–85. 26120806 10.1016/j.jad.2015.05.062

[18] Jendricko T, Vidovic A, Grubisic-Ilic M, Romic Z, Kovacic Z, Kozaric-Kovacic D. Homocysteine and serum lipids concentration in male war veterans with posttraumatic stress disorder. Prog Neuropsychopharmacol Biol Psychiatry. 2009;33:134–40. 19038303 10.1016/j.pnpbp.2008.11.002

[19] Kuebler U, Linnebank M, Semmler A, Stoffel-Wagner B, La Marca R, Ehlert U, et al. Plasma homocysteine levels increase following stress in older but not younger men. Psychoneuroendocrinology. 2013;38:1381–7. 23312061 10.1016/j.psyneuen.2012.12.003

[20] Chung KH, Chiou HY, Chen YH. Associations between serum homocysteine levels and anxiety and depression among children and adolescents in Taiwan. Sci Rep.2017;7:8330. 28827592 10.1038/s41598-017-08568-9 PMC5566365

[21] Fraguas R Jr, Papakostas GI, Mischoulon D, Bottiglieri T, Alpert J, Fava M. Anger attacks in major depressive disorder and serum levels of homocysteine. Biol Psychiatry. 2006;60:270–4. 16325154 10.1016/j.biopsych.2005.08.026

[22] Saraswathy KN, Ansari SN, Kaur G, Joshi PC, Chandel S. Association of vitamin B12 mediated hyperhomocysteinemia with depression and anxiety disorder: a cross-sectional study among Bhil indigenous population of India. Clin Nutr Espen. 2019;30:199–203. 30904222 10.1016/j.clnesp.2019.01.009

[23] Moradi F, Lotfi K, Armin M, Clark CC, Askari G, Rouhani MH. The association between serum homocysteine and depression: a systematic review and meta‐analysis of observational studies. Eur J Clin Investig. 2021;51:e13486. 33423269 10.1111/eci.13486

[24] Chung K-H, Chiou H-Y, Chen Y-H. Associations between serum homocysteine levels and anxiety and depression among children and adolescents in Taiwan. Sci Rep. 2017;7:8330. 28827592 10.1038/s41598-017-08568-9 PMC5566365

[25] Evans MK, Lepkowski JM, Powe NR, LaVeist T, Kuczmarski MF, Zonderman AB. Healthy aging in neighborhoods of diversity across the life span (HANDLS): overcoming barriers to implementing a longitudinal, epidemiologic, urban study of health, race, and socioeconomic status. Ethn Dis. 2010;20:267–75. 20828101 PMC3040595

[26] Suen KFK, Lee GR, Finnegan M, Halton K, Borovickova I, Trench C, et al. Total plasma homocysteine measurement: evaluation of the Abbott immunoassay, comparison with the JEOL ion exchange chromatography and investigation of its clinical utility. Pr Lab Med. 2022;32:e00295. 10.1016/j.plabm.2022.e00295 PMC9386494 35992628

[27] Jones B, Nagin D, Roeder K. A SAS procedure based on mixture models for estimating developmental trajectories. Sociol Methods Res. 2001;29:374–93.

[28] Jones B, Nagin D. Advances in group-based trajectory modeling and an SAS procedure for estimating them. Sociol Method Res. 2007;35:542–71.

[29] Nguyen HT, Kitner-Triolo M, Evans MK, Zonderman AB. Factorial invariance of the CES-D in low socioeconomic status African Americans compared with a nationally representative sample. Psychiatry Res. 2004;126:177–87. 15123397 10.1016/j.psychres.2004.02.004

[30] Beekman AT, Deeg DJ, Van Limbeek J, Braam AW, De Vries MZ, Van Tilburg W. Criterion validity of the Center for Epidemiologic Studies Depression scale (CES-D): results from a community-based sample of older subjects in The Netherlands. Psychol Med. 1997;27:231–5. 9122304 10.1017/s0033291796003510

[31] Beydoun MA, Obhi HK, Weiss J, Canas JA, Beydoun HA, Evans MK, et al. Systemic inflammation is associated with depressive symptoms differentially by sex and race: a longitudinal study of urban adults. Mol Psychiatry. 2020;25:1286–1300. 31019266 10.1038/s41380-019-0408-2 PMC6813878

[32] Zimmerman M, Chelminski I. Screening for anxiety disorders in depressed patients. J Psychiatr Res. 2006;40:267–72. 15893327 10.1016/j.jpsychires.2005.03.001

[33] Gibbons RD, Rush AJ, Immekus JC. On the psychometric validity of the domains of the PDSQ: an illustration of the bi-factor item response theory model. J Psychiatr Res. 2009;43:401–10. 18554611 10.1016/j.jpsychires.2008.04.013

[34] Annual Update of the HHS Poverty Guidelines; Notice. https://aspe.hhs.gov/sites/default/files/documents/ff1ac67af87462a031279a1a462bcd13/HHS-Poverty-Guidelines-Fed-Register-2004.pdf, Date Accessed 2004 Accessed.

[35] Beydoun HA, Huang S, Beydoun MA, Hossain S, Zonderman AB. Mediating-moderating effect of allostatic load on the association between dietary approaches to stop hypertension diet and all-cause and cause-specific mortality: 2001-2010 National Health and Nutrition Examination Surveys. Nutrients. 2019;11:503. 31569527 10.3390/nu11102311 PMC6836046

[36] Beydoun MA, Beydoun HA, Mode N, Dore GA, Canas JA, Eid SM, et al. Racial disparities in adult all-cause and cause-specific mortality among us adults: mediating and moderating factors. BMC Public Health. 2016;16:1113. 27770781 10.1186/s12889-016-3744-z PMC5075398

[37] Beydoun MA, Beydoun HA, Dore GA, Canas JA, Fanelli-Kuczmarski MT, Evans MK, et al. White blood cell inflammatory markers are associated with depressive symptoms in a longitudinal study of urban adults. Transl Psychiatry. 2016;6:e895. 27648917 10.1038/tp.2016.180 PMC5048214

[38] Beydoun MA, Beydoun HA, Dore GA, Fanelli-Kuczmarski MT, Evans MK, Zonderman AB. Total serum cholesterol, atherogenic indices and their longitudinal association with depressive symptoms among US adults. Transl Psychiatry. 2015;5:e518. 25734511 10.1038/tp.2015.4 PMC4354360

[39] Beydoun MA, Hossain S, Chitrala KN, Tajuddin SM, Beydoun HA, Evans MK, et al. Association between epigenetic age acceleration and depressive symptoms in a prospective cohort study of urban-dwelling adults. J Affect Disord. 2019;257:64–73. 31299406 10.1016/j.jad.2019.06.032 PMC6757325

[40] Beydoun MA, Beydoun HA, Kitner-Triolo MH, Kaufman JS, Evans MK, Zonderman AB. Thyroid hormones are associated with cognitive function: moderation by sex, race, and depressive symptoms. J Clin Endocrinol Metab. 2013;98:3470–81. 23690311 10.1210/jc.2013-1813 PMC3733856

[41] Selvin S. Statistical Analysis of Epidemiologic Data. 3rd edn. Oxford University Press, 2004.

[42] Hochberg Y, Tamhane, AC. Multiple comparison procedures. Wiley: New York, 1987.

[43] Beydoun MA, Canas JA, Dore GA, Beydoun HA, Rostant OS, Fanelli-Kuczmarski MT, et al. Serum uric acid and its association with longitudinal cognitive change among urban adults. J Alzheimers Dis. 2016;52:1415–30. 27104899 10.3233/JAD-160028 PMC4902772

[44] Bremner JD, Goldberg J, Vaccarino V. Plasma homocysteine concentrations and depression: a twin study. J Affect Disord Rep. 2021;4:100093. 10.1016/j.jadr.2021.100087 PMC8372975 34414386

[45] Castro F, Melgarejo J, Chavez CA, de Erausquin GA, Terwilliger JD, Lee JH, et al. Total plasma homocysteine and depressive symptoms in older hispanics. J Alzheimers Dis. 2021;82:S263–9. 33579837 10.3233/JAD-201062 PMC8300858

[46] Zhou H, Zhong X, Chen B, Wu Z, Zhang M, Mai N, et al. Interactive effects of elevated homocysteine and late-life depression on cognitive impairment. J Affect Disord. 2020;277:212–7. 32829197 10.1016/j.jad.2020.08.022

[47] Ford AH, Flicker L, Singh U, Hirani V, Almeida OP. Homocysteine, depression and cognitive function in older adults. J Affect Disord. 2013;151:646–51. 23928176 10.1016/j.jad.2013.07.012

[48] Forti P, Rietti E, Pisacane N, Olivelli V, Dalmonte E, Mecocci P, et al. Blood homocysteine and risk of depression in the elderly. Arch Gerontol Geriatr. 2010;51:21–25. 19646770 10.1016/j.archger.2009.06.009

[49] Beydoun MA, Tajuddin SM, Shaked D, Beydoun HA, Evans MK, Zonderman AB. One-carbon metabolism gene polymorphisms are associated with cognitive trajectory among African-American adults. Neurobiol Aging. 2019;84:70–82. 31208817 10.1016/j.neurobiolaging.2019.05.013 PMC12264804

[50] Watkins D, Ru M, Hwang HY, Kim CD, Murray A, Philip NS, et al. Hyperhomocysteinemia due to methionine synthase deficiency, cblG:: structure of the gene, genotype diversity, and recognition of a common mutation, P1173L. Am J Hum Genet. 2002;71:143–53. 12068375 10.1086/341354 PMC384971

[51] Pawlak R. Is vitamin B12 deficiency a risk factor for cardiovascular disease in vegetarians? Am J Prev Med. 2015;48:e11–26. 25998928 10.1016/j.amepre.2015.02.009

[52] Zaghloul A, Iorgoveanu C, Desai A, Balakumaran K, Chen K. Methylenetetrahydrofolate reductase polymorphism and premature coronary artery disease. Cureus. 2019;11:e5014. 31497444 10.7759/cureus.5014 PMC6716763

[53] Jadavji NM, Wieske F, Dirnagl U, Winter C. Methylenetetrahydrofolate reductase deficiency alters levels of glutamate and gamma-aminobutyric acid in brain tissue. Mol Genet Metab Rep. 2015;3:1–4. 26937386 10.1016/j.ymgmr.2015.02.001 PMC4750636

[54] Bhatia P, Singh N. Homocysteine excess: delineating the possible mechanism of neurotoxicity and depression. Fundam Clin Pharm. 2015;29:522–8. 10.1111/fcp.12145 26376956

[55] Lee ES, Chen H, Soliman KF, Charlton CG. Effects of homocysteine on the dopaminergic system and behavior in rodents. Neurotoxicology. 2005;26:361–71. 15935208 10.1016/j.neuro.2005.01.008

[56] Beydoun MA, Weiss J, Obhi HK, Beydoun HA, Dore GA, Liang H, et al. Cytokines are associated with longitudinal changes in cognitive performance among urban adults. Brain Behav Immun. 2019;80:474–487. 30981715 10.1016/j.bbi.2019.04.027 PMC6698146

