# Supplementary material for: Longitudinal association of homocysteine with depressive and anxiety symptoms among urban adults: healthy aging in neighborhoods of diversity across the life span study
Source: Transl Psychiatry. 2024 Oct 19;14:444. doi: 10.1038/s41398-024-03111-7 (PMC11490487; doi:10.1038/s41398-024-03111-7)
Supplement: Supplementary file 1 — Online Supplementary Materials [file 41398_2024_3111_MOESM1_ESM.pdf]

Supplemental Materials

Supplemental Methods 1 –Anxiety Items

Wave	File	Variable name	Variable label	Factor levels
1	ACASI	PDSQga01	ACASI189: Were you a nervous person on most days	No|Yes|DK|NA
1	ACASI	PDSQga02	ACASI190: Worry that bad things might happen to you or someone close to you	No|Yes|DK|NA
1	ACASI	PDSQga03	ACASI191: Worry about things that other people said shouldn`t worry you	No|Yes|DK|NA
1	ACASI	PDSQga04	ACASI192: Worried or anxious about your daily life on most days	No|Yes|DK|NA
1	ACASI	PDSQga05	ACASI193: Feel restless or on edge because you were worrying	No|Yes|DK|NA
1	ACASI	PDSQga06	ACASI194: Problems falling asleep because you were worrying about things	No|Yes|DK|NA
1	ACASI	PDSQga07	ACASI195: Tension in your muscles because of anxiety or stress	No|Yes|DK|NA
1	ACASI	PDSQga08	ACASI196: Difficulty concentrating because your mind was on your worries	No|Yes|DK|NA
1	ACASI	PDSQga09	ACASI197: Snappy or irritable because you were worrying or feeling stressed	No|Yes|DK|NA
1	ACASI	PDSQga10	ACASI198: Hard for you to control or stop your worrying on most days	No|Yes|DK|NA

Supplemental Methods 2 – Mixed-effects linear regression models and missing outcome data handling in *mixed* and *traj* commands:

2.1. Mixed-effects linear regression models[1, 2]

The main multiple mixed-effects regression models can be summarized as follows:

Multi-level models vs. Composite models

Eq. 1.1-1.4

Multi-level models		Composite models
$Y_{ij} = \pi_{0i} + \pi_{1i}Time_{ij} + \varepsilon_{ij}$	$\pi_{0i} = \gamma_{00} + \gamma_{0a}X_{aij} + \sum_{k=1}^{l}\gamma_{0k}Z_{ik} + \zeta_{0i}$ $\pi_{1i} = \gamma_{10} + \gamma_{1a}X_{aij} + \sum_{m=1}^{n}\gamma_{1m}Z_{im} + \zeta_{1i}$	$Y_{ij} = \gamma_{00} + \gamma_{0a}X_{aij} + \sum_{k=1}^{l}\gamma_{0k}Z_{ik}$ $+ \gamma_{10}Time_{ij} + \gamma_{1a}X_{aij}Time_{ij}$ $+ \sum_{m=1}^{n}\gamma_{1m}Z_{im}Time_{ij}$ $+ (\zeta_{0i} + \zeta_{1i}Time_{ij} + \varepsilon_{ij})$

Where Y_{ij} is the outcome (5 depressive symptoms test scores measured at v_1, v_2, and/or v_3) for each individual "i" and visit "j"; π_{0i} is the level-1 intercept for individual i; π_{1i} is the level-1 slope for individual i; γ_{00} is the level-2 intercept of the random intercept π_{0i}; γ_{10} is the level-2 intercept of the slope π_{1i}; Z_{ik} is a vector of fixed covariates for each individual *i* that are used to predict level-1 intercepts and slopes, which can include socio-demographic variables among others. In this analysis, mixed-effects regression models included HCY exposure measured at v_1 or as a trajectory exposure (Probability of belonging to "High increasing" group, z-scored) (X_{ij}), along with covariates (Z_{ij}). ζ_{0i} and ζ_{1i} are level-2 disturbances; ε_{ij} is the within-person level-1 disturbance [2].

It is worth noting that the models were fit using the entire HANDLS study cohort with complete data on either v_1, v_2 or v_3 for each depressive symptoms test score, those models

were used to improve reliability of predicted estimates. Empirical Bayes estimators for annual rate of change in each depressive symptoms test score were also predicted from time-interval mixed-effects models, with up to 3 repeats on each outcome, without adding any covariates in the model aside from *TIME*.

2.2. Group-based trajectory models[3, 4]

The *traj* function in Stata allows for the application of group-based trajectory models, which are a robust method for identifying and assessing unique developmental trajectories within a population. By applying these models, researchers can acquire a more profound comprehension of the variability in longitudinal data and the diverse patterns that exist within their study population. Trajectories refer to the patterns that individuals follow over time in relation to a specific outcome. For instance, in a study on academic achievement, the trajectory could depict the fluctuations in test scores over a span of multiple years. GBTM operates under the assumption that the population consists of discrete groups of individuals, each of which follows a similar path. The objective is to identify these groups and delineate their distinct trajectories. GBTM recognizes the presence of diversity within the population. GBTM acknowledges the possibility of numerous different trajectories, rather than assuming that all individuals follow the same trajectory or that individual trajectories vary around a single mean trajectory. Modeling strategies involve deciding on the number of trajectories and their form (linear, quadratic, cubic) over time or age, and finding the best fitting model using among others, the Bayesian Information Criterion to compare among alternative models. In addition, a minimum membership probability per group may be set a prior. In the present study, this minimum was 10%.

2.3. Handling of missing outcome data in mixed and traj commands

2.3.1. Stata mixed command [1, 2]:

The *mixed* command in Stata is utilized to fit mixed-effects models, which are very valuable in the examination of longitudinal data. The mixed command utilizes a special method when handling missing data on the outcome variable.

Listwise Deletion, also known as Complete Case Analysis, is a method used in Stata to handle missing data on the result variable. It involves removing any observations that have missing values on the outcome variable. Consequently, every instance (i.e., any entry in the dataset) that lacks a value for the outcome variable will be omitted from the analysis. Stata automatically performs this task without incorporating any missing data into the estimation procedure.

Effect on Random Effects: The random effects structure is influenced by missing data in the result variable. The contribution of each subject to the likelihood is computed by considering the outcome data that is accessible and not missing for that subject. When there is missing data for a subject at specific time points, only the data points that were actually seen for that subject will be used to estimate the random effects.

The *mixed* command is particularly effective for managing unbalanced data, a common occurrence in longitudinal research where participants may have varying amounts of repeated assessments due to missing outcomes. The mixed-effects model can include a subject in the analysis as long as there is at least one non-missing observation for that subject.

To summarize, the *mixed* command in Stata handles missing data on the outcome variable by removing any observations with missing outcomes from the analysis. This method streamlines the management of

missing data, but it can result in data loss and associated biases if the absence of data is not entirely random.

2.3.2. Traj command[3, 4]

The traj function in Stata is utilized to estimate group-based trajectory models, which aim to detect clusters of individuals exhibiting similar patterns of change across time[23, 24]. The traj command utilizes a distinct methodology in managing missing data, in contrast to the mixed command. Here is the approach it takes when dealing with missing data:

The traj command use maximum likelihood estimation (MLE) to address missing data on the result variable. This implies that it includes all existing data points without excluding observations completely even if part of their data points are absent. The estimating procedure incorporates the existing data and utilizes it to calculate the model parameters.

The Maximum Likelihood Estimation (MLE) approach implies that the missing data follows the Assumption of Missing at Random (MAR). Given this premise, the probability of the observed data can be accurately assessed without any prejudice, as long as the chance of missing data is only dependent on the observed data and not on the unobserved data.

Partial Data Utilization: In contrast to listwise deletion, which would exclude any individual with any missing data point, the traj command allows for the inclusion of individuals with partially missing data. When a subject has missing time points, but not all, the trajectory modeling still utilizes the available data points for that subject.

The strategy provides enhanced flexibility in dealing with missing data, particularly in longitudinal research where the occurrence of missing data is frequent. The traj command can often yield more robust

and generalizable results by not eliminating entire cases based on lacking observations.

In summary, the traj command in Stata deals with missing data by employing maximum likelihood estimation. This method enables the command to utilize all available data points, while assuming that the missing data is randomly distributed. This methodology aids in preserving a greater amount of data during the analysis process and mitigates the potential biases that might arise from listwise elimination.

Table S1. LnOdds of elevated anxiety probabilities vs. LnHcy at baseline: OLS linear regression models; HANDLS 2004-2017

	**Model 1**			**Model 2**		
	**β**	**SE**	**P**	**β**	**SE**	**P**
Main predictor						
**LnHcy**	-0.18	0.68	0.792	-0.37	0.68	0.585
Covariates						
**Age**	**-0.07**	**0.02**	**0.005**	**-0.06**	**0.02**	**0.018**
**Sex**	**-1.71**	**0.45**	**<0.001**	**-2.06**	**0.45**	**<0.001**
**Race**	**-1.91**	**0.43**	**<0.001**	**-1.97**	**0.43**	**<0.001**
**Poverty Status**	**+1.89**	**0.45**	**<0.001**	**+1.08**	**0.46**	**0.019**

Abbreviations: HANDLS=Health Aging in Neighborhoods of Diversity Across the Life Span; Hcy=Homocysteine; Ln=Loge transformed.

Model 1 is adjusted for age, sex, race, poverty status and the inverse mills ratio; Model 2 is Model 1 further adjusted for education, current smoking, current drug use, HEI-2010 total score and the body mass index, all measured at visit 1. Only covariates included in both models are shown. Sex: 1=Women, 2=Men; Race: 1=White, 2=African American; Poverty status: 1=Above Poverty, 2=Below Poverty.

Table S2. Relationship of homocysteine annualized change between v1 and v2, z-scored (Hcy_{ch}) with depressive symptoms total and domain-specific scores (baseline and between-visit change), overall: Mixed-effects linear regression models, HANDLS 2004-2017

	**Hcy_{ch}:** Annualized change in LnHcy, z-scored			
	Model 1 [1]		Model 2 [2]	
	β (SE)	P	β (SE)	P
**OVERALL [3]**				
CES-D total score:	N=1,457, K=2.8		N=1,457, K=2.8	
Hcy_{ch}	**+0.742 (0.285)**	**0.009**	**+0.640 (0.278)**	**0.021**
Hcy_{ch} × Time	-0.021 (0.034)	0.535	-0.022 (0.034)	0.518
CES-D domain 1 score:	N=1,457, K=2.8		N=1,457, K=2.8	
Hcy_{ch}	**+0.258 (0.125)**	**0.039**	+0.224 (0.123)	0.068
Hcy_{ch} × Time	-0.010 (0.015)	0.492	-0.010 (0.015)	0.504
CES-D domain 2 score:	N=1,457, K=2.8		N=1,457, K=2.8	
Hcy_{ch}	**+0.075 (0.033)**	**0.025**	**+0.069 (0.033)**	**0.038**
Hcy_{ch} × Time	-0.006 (0.005)	0.161	-0.006 (0.005)	0.164
CES-D domain 3 score:	N=1,457, K=2.8		N=1,457, K=2.8	
Hcy_{ch}	**+0.311 (0.112)**	**0.006**	**+0.265 (0.110)**	**0.016**
Hcy_{ch} × Time	-0.001 (0.015)	0.948	-0.001 (0.015)	0.924
CES-D domain 4 score:	N=1,457, K=2.8		N=1,457, K=2.8	
Hcy_{ch}	-0.077 (0.066)	0.246	-0.059 (0.066)	0.371
Hcy_{ch} × Time	+0.001 (0.010)	0.900	+0.002 (0.010)	0.848

Abbreviations: CES-D = Center for Epidemiological Studies Depression; Hcy_{ch}= z-transformed of annualized change in LnHcy, z-scored; K = Mean number of visits per subject; N = Sample size; SE = Standard error. [1] Model 1 is adjusted for age, sex, race, poverty status, inverse mills ratio as well as time on study in years between visits 1 and 3 and its interaction with homocysteine trajectory and covariates. [2] Model 2 is adjusted for age, sex, race, poverty status, education, literacy, smoking, drug use, 2010 healthy eating index, body mass index, inverse mills ratio as well as time on study in years between visits 1 and 3 and its interaction with homocysteine trajectory and covariates. [3] Depressive symptoms scores include the CES-D total score, the CES-D domain 1 score [depressive affect], the CES-D domain 2 score [interpersonal problems], the CES-D domain 3 score [somatic complaints] and the CES-D domain 4 score [positive affect].

Table S3. Depressive and anxiety symptoms as predictors of $\log_e$ transformed homocysteine at baseline and over time: mixed-effects linear regression models, HANDLS 2004-2017

	Model 1 [1]		Model 2 [2]	
	β (SE)	P value	β (SE)	P value
w1CEScenter16:	N=1,445, K=2.8		N=1,445, K= 2.8	
w1CEScenter16	**+0.001961 (0.000740)**	**0.010**	**+0.001583 (0.000757)**	**0.037**
w1CEScenter16 × Time	+0.000046 (0.000087)	0.598	+0.000000 (0.000090)	0.985
w1ANXIETY_ORD:	N= 1,181, K= 2.8		N= 1,181, K=2.8	
w1ANXIETY_ORD	0.004279 (0.002931)	0.144	0.003189 (0.002955)	0.280
w1ANXIETY_ORD × Time	0.000343 (0.000333)	0.303	0.000288 (0.000338)	0.395
zR_traj_ProbG2ANXIETY:	N= 1,383, K=2.8		N=1,383, K=2.8	
zR_traj_ProbG2ANXIETY	0.002406 (0.008502)	0.777	-0.001235 (0.0086)	0.886
zR_traj_ProbG2ANXIETY × Time	**+0.001904 (0.000956)**	**0.046**	+0.001568 (0.000975)	0.108
w1AnxietyDisorder:	N=1,346, K=2.8		N=1,346, K=2.8	
w1AnxietyDisorder	-0.005374 (0.027987)	0.848	-0.014071 (0.028 048)	0.616
w1AnxietyDisorder × Time	0.00357 (0.003438)	0.299	0.003117 (0.003473)	0.369

Abbreviations: CES-D = Center for Epidemiological Studies Depression; Hcy = Homocysteine; K = Mean number of visits per subject; N = Sample size; SE = Standard error; w1CEScenter16=wave 1 (or visit 1) CES-D total score centered at 16; w1ANXIETY_ORD=wave 1 (i.e. visit 1) anxiety total score, ordinal scale; w1AnxietyDisorder=wave 1 (i.e. visit 1) anxiety disorder, yes vs. no, based no self-report; zR_traj_ProbG2ANXIETY=Probability of group 2 anxiety trajectory, z-scored.

[1] Model 1 is adjusted for age, sex, race, poverty status, inverse mills ratio as well as time on study between visits 1 and 2 (in years) and its interaction with homocysteine and covariates. [2] Model 2 is adjusted for age, sex, race, poverty status, education, smoking, drug use, 2010 healthy eating index, body mass index, inverse mills ratio as well as time on study between visits 1 and 2 (in years) and its interaction with homocysteine and covariates.

Table S4. Baseline Hcy and Hcy trajectory exposures as predictors for incident elevated depressive symptoms over time (CES-D total score≥16): Cox proportional hazards model, HANDLS 2004-2017[1]

	**Baseline Hcy exposure vs. incident EDS**			**Hcy_{traj} exposure vs. incident EDS**		
	HR	95% CI	P	HR	95% CI	P
**Overall**	1.06	0.89-1.26	0.522	**1.09**	**1.03-1.14**	**0.001**
**Women**	1.12	0.90-1.39	0.306	**1.14**	**1.06-1.22[2]**	**<0.001**
**Men**	0.87	0.64-1.17	0.348	1.01	0.94-1.09[2]	0.728
**White**	**0.50**	**0.37-0.67[2]**	**<0.001**	1.03	0.93-1.13	0.607
**African American**	**1.49**	**1.24-1.81[2]**	**<0.001**	**1.10**	**1.03-1.16**	**0.003**
**Above Poverty**	**0.75**	**0.59-0.95[2]**	**0.018**	1.02	0.95-1.09[2]	0.581
**Below Poverty**	**1.60**	**1.25-2.03[2]**	**<0.001**	**1.22**	**1.13-1.32[2]**	**<0.001**
**Below median Anxiety**	**1.62**	**1.31-2.01[2]**	**<0.001**	**1.09**	**1.02-1.18**	**0.010**
**Above median Anxiety**	1.22	0.92-1.60[2]	0.167	1.05	0.96-1.15	0.325

Abbreviations: CES-D = Center for Epidemiological Studies Depression; CI=Confidence Interval; EDS=Elevated Depressive Symptoms; HR=Hazard Ratio; N = Sample size; SE = Standard error. [1]

[1]All models were adjusted for age, sex, race, poverty status, education, literacy, smoking, drug use, 2010 healthy eating index, body mass index, inverse mills ratio. See Methods section for full description of the two main exposures.

[2] $P<0.05$ for null hypothesis of $\gamma=0$, where γ is the 2-way interaction parameter in the fully adjusted unstratified model between sex, race, poverty status and anxiety score level and each of the two Hcy exposures. Two-way interaction terms were tested separately for each potential effect modifier.

REFERENCES:

1. Rabe-Hesketh S, & Skrondal, A.,. *Multilevel and Longitudinal Modeling Using Stata*, 2012.

2. Blackwell E, de Leon CF, Miller GE. Applying mixed regression models to the analysis of repeated-measures data in psychosomatic medicine. *Psychosom Med* 2006; **68**(6)**:** 870-878.

3. Jones B, Nagin D, Roeder K. A SAS procedure based on mixture models for estimating developmental trajectories. *Sociological Methods & Research* 2001; **29:** 374-393.

4. Jones B, Nagin D. Advances in group-based trajectory modeling and an SAS procedure for estimating them. *Sociological Methods & Research* 2007; **35:** 542-571.
